# Transfusion Requirements in Surgical Oncology Patients (TRISOP): a randomized, controlled trial

**DOI:** 10.1186/cc12302

**Published:** 2013-03-19

**Authors:** J Almeida, F Galas, E Osawa, J Fukushima, S Moulin, C Park, E Almeida, S Vieira, J Vincent, A Rhodes, M Balzan, J Inacio, H Palomba, R Nakamura, F Bergamin, A Sandrini, U Ribeiro Jr, J Auler Jr, L Hajjar

**Affiliations:** 1 Instituto do Cancer do Estado de São Paulo, Brazil; 2 Erasme Hospital, Université libre de Bruxelles, Brussels, Belgium; 3 St George's Healthcare NHS Trust and St George's University of London, UK

## Introduction

The purpose of this study was to evaluate whether a restrictive strategy of red blood cell (RBC) transfusion was superior to a liberal one for reducing mortality and severe clinical complications among patients undergoing major cancer surgery.

## Methods

The trial was designed as a phase III, randomized, controlled, parallel-group, superiority trial. The inclusion criteria were adult patients with cancer who were undergoing major abdominal surgery requiring postoperative care in an ICU. The patients were randomly allocated to treatment with either a liberal RBC transfusion strategy (transfusion when hemoglobin levels decreased below 9 g/dl) or a restrictive RBC transfusion strategy (transfusion when hemoglobin levels decreased below 7 g/dl). The primary outcome was a composite endpoint of death or severe complications. The patients were monitored for 30 days.

## Results

A total of 1,521 patients were screened for eligibility and 248 met the inclusion criteria. After exclusions for medical reasons or a lack of consent, 198 patients were included in final analysis, with 101 allocated to the restrictive group and 97 to the liberal group. The primary composite endpoint - all-cause mortality, cardiovascular complications, acute respiratory distress syndrome, acute kidney injury requiring renal replacement therapy, septic shock or reoperation at 30 days - occurred in 19.6% of the patients in the liberal strategy group and in 35.6% in the restrictive group (*P *= 0.012). The liberal strategy group had a significantly lower 30-day mortality rate as compared with the restrictive group (8.2% (95% CI, 4.2 to 15.4%) vs. 22.8% (95% CI, 15.7 to 31.9%), respectively, *P *= 0.005). The occurrence of cardiovascular complications was lower in the liberal group than in the restrictive group (5.2% (95% CI, 2.2 to 11.5%) vs. 13.9% (95% CI, 8.4 to 21.9%), respectively, *P *= 0.038). The restrictive strategy group had a higher 60 day mortality rate as compared with the liberal group (23.8% (95% CI, 16.5 to 32.9%) vs. 11.3% (95% CI, 6.5 to 9.2%), respectively, *P *= 0.022). 

## Conclusion

The liberal RBC transfusion strategy with a hemoglobin trigger of 9 g/dl was associated with fewer major postoperative complications in patients undergoing major cancer surgery compared with the restrictive strategy.

